# Assessment of key interviewing factors for research assistants (AKIRA): development of a novel training and evaluation competency-based tool for mental health data collection in community settings

**DOI:** 10.3389/feduc.2025.1539124

**Published:** 2025-03-12

**Authors:** Alejandra Cid-Vega, Chynere Best, Kendall Pfeffer, Manaswi Sangraula, Janus Wong, Wilfred Gwaikolo, James Caracoglia, Sauharda Rai, Adam D. Brown, Brandon Kohrt

**Affiliations:** 1 Department of Psychology, The New School for Social Research, New York, NY, United States; 2 Department of Psychology, University of Miami, Coral Gables, FL, United States; 3 Department of Psychiatry and Behavioral Health, Center for Global Mental Health Equity, George Washington University, Washington, DC, United States; 4 World Trade Center Mental Health Program, Icahn School of Medicine at Mount Sinai, New York, NY, United States; 5 Department of Psychiatry, New York University School of Medicine, New York, NY, United States

**Keywords:** mental health research, monitoring and evaluation, competency assessment, global mental health, cross-cultural, capacity building, equity

## Abstract

Data quality is critical in mental health research, but variability in training among those collecting data can undermine research outcomes. In this context, the Assessment of Key Interviewing Factors for Research Assistants (AKIRA) emerges as a novel, competency-based framework specifically designed for interview-based mental health data collection. AKIRA systematically identifies and evaluates key interviewing behaviors across ten domains, highlighting areas of mastery, improvement, and potentially harmful practices. Emphasizing cross-cultural applicability, the tool adapts to diverse research settings, particularly where non-specialist data collectors play central roles. Global mental health services face significant challenges, with treatment rates for conditions such as depression alarmingly low, especially in lower-to-middle income countries. Such disparities underscore the urgent need for evidence-based, culturally sensitive interventions and robust monitoring systems to bridge gaps in mental health care. Despite the growing demand for high-quality data, there is a marked absence of systematic competency assessments for research assistants, contributing to variability and potential bias in data collection processes. AKIRA was developed through an iterative process involving literature reviews, adaptation of existing frameworks for competency assessment, and feedback from key informants. Its pilot testing and ongoing evaluation aim to refine its utility, ensuring that non-specialist data collectors are better prepared to engage with communities and conduct reliable, replicable research. By standardizing interview techniques and addressing the “interviewer effect,” AKIRA not only enhances data quality but also facilitates ethical, culturally informed research practices. Future psychometric evaluations and cross-context adaptations, including implementations in the United States, Uganda, and Nepal, promise to further integrate this tool into mental health research infrastructures, ultimately supporting more effective program monitoring and improved mental health outcomes globally. Overall, AKIRA represents a transformative step in standardizing data collection competencies. Its broad adoption could enhance research quality, inform policy decisions, and ultimately contribute to reducing global mental health disparities at scale.

Data quality is critical in mental health research, yet there is considerable variability in the training and preparation of those involved in data collection. Individuals with limited research training often play a central role in collecting data and, to date, no systematic means of evaluating researcher competencies exists. The Assessment of Key Interviewing factors for Research Assistants (AKIRA), a competency-based tool for researchers conducting interview-based mental health data collection with communities, aims to address this gap. AKIRA provides a systematic framework for the training, monitoring and evaluation of data collectors, by identifying key interviewing behaviors across 10 domains, and assessing research assistants for mastery, areas for improvement, and displays of harmful behaviors in these domains. With an emphasis on cross-cultural interactions, the AKIRA is designed to be easily adapted to various implementation contexts. Given the information gaps in mental health research, and the importance of monitoring and evaluation in the development and guidance of mental health interventions, AKIRA may enhance data quality and research especially in community mental health settings where the experiences and training of individuals involved in research and data collections may vary considerably and would benefit from competency-based trainings to support this complex work.

A major service gap exists globally where individuals’ and communities’ mental health needs far exceed the availability of specialized care. For instance, only 23% of individuals with depression receive adequate treatment in high-income countries, a rate that drops to 3% for populations receiving treatment for depression in lower-to-middle income countries (LIMCs) (World Health Organization, 2022). Addressing these service gaps in mental health throughout the world requires the development, cultural and contextual adaptation, implementation, monitoring, and evaluation of evidence-based interventions (Jordans and Brandon, 2020; Malekzadeh et al., 2020; Wainberg et al., 2017; World Health Organization, 2022). The World Health Organization (2022) recently emphasized the need for greater integration of research and evaluation into existing programs as a critical goal for reducing gaps in equity and improving care, yet the generation of solid evidence through research for mental health interventions continues to remain a challenge globally. Published, peer-reviewed research on various domains in existing programs is sparse in areas such as reach, effectiveness, barriers and facilitators, or impact among others. These gaps may be influenced by a wide range of factors such as resource gaps, training variations in data collection processes, and the complex contexts in which programs are being delivered (Thornicroft et al., 2017; World Health Organization, 2022; Patel et al., 2018). For instance, although 82% of the nearly one billion people with a mental health disorder live in LIMCs (World Health Organization, 2022), only 3.57% of health-related publications and 6% of open access literature on mental health derive from these contexts (Saxena et al., 2006). Moreover, despite the urgent public health concern that mental health challenges pose for communities around the world, mental health care often accounts for only 2% of national budgets (World Health Organization, 2022). Thus, a stronger evidence base is necessary to shape mental health programming, policy and investments in early prevention and healthcare systems to feasibly, effectively address the culturally unique needs of a given community or setting (Collins et al., 2011; Proctor et al., 2009).

Among the challenges and barriers that exist for carrying out research, especially in lower resourced or emergency settings, is the preparedness of individuals to collect data related to mental health programs. The lack of data collector readiness may result in decreased data collection activities and potential loss of important data, particularly from under-resourced communities, needed for a reliable, replicable and generalizable evidence base (Hossen, 2022). Several reasons may account for gaps in research and monitoring and evaluation carried out in mental health programs globally. Among mental health programs being delivered throughout the world, little is reported about the standards and consistency in which data is collected and the extent to which those involved have received training in data collection processes (Macfarlane and Carla, 2019). Additionally, the extent to which mental health programs consider methodological designs as part of their implementation strategies remains largely unknown. Furthermore, stakeholders, such as non-profit and community-based organizations are often funded to deliver mental health interventions with little to no support dedicated to research or monitoring and evaluation. As such, the resources needed to carry out these efforts may be perceived as an additional burden for which staff may not be adequately supported.

Moreover, variability in data collector competency may discourage lack of participant engagement in research or bias in data collected. Among individuals receiving mental health services, data collection challenges may also exist due to mistrust in research processes (Holden et al., 2015; Cokley and Awad, 2013; Singh et al., 2018). Underrepresentation of ethnic minorities and other minoritized groups in research reduces the ability to generalize findings and may widen the gap in health inequities (Waheed et al., 2015). Mistrust, stigma around research, and the scarcity of culturally sensitive researchers are major deterrents to willing participants (Holden et al., 2015; Singh et al., 2018). Such mistrust derives in part from histories of exploitation and deceit in which minoritized and vulnerable communities were either forced to participate in studies, or included in research without adequate information and consent (Cokley and Awad, 2013; Singh et al., 2018). Importantly, an explicit commitment to social justice, cultural sensitivity and more inclusive data collection in research practices, is critical to foster more equitable approaches to research and information that captures a greater diversity of lived experiences (Cokley and Awad, 2013).

Competency training, particularly at a cross-cultural level, linguistic adaptation, capacity building and frameworks that aid in the standardization of participant-researcher interactions, are pivotal next steps toward developing a strong evidence base reflective of the contexts in which work is carried out. The “interviewer effect” describes the potential bias introduced when respondents’ perceptions of the interviewer influence an answer, resulting in potential measurement errors, particularly found in data collection processes in public health research (Davis et al., 2010). This highlights the importance of training interviewers in cross-cultural data collection among other critical interpersonal and professional competencies. Furthermore, there is a prominent gap in availability of training addressing data collection practices in addition to the standardization of frameworks to conduct these processes, particularly in lower-to-middle income contexts and community settings (Holden et al., 2015; Wainberg et al., 2017; Collins and Beverly, 2016). Capacity building in mental health research from the level of field data collection requires a targeted strategy that begins to bridge research, development, implementation, and policy (Wainberg et al., 2017; Collins and Beverly, 2016).

Despite the many factors that contribute to information gaps across global and community mental health programs, one potential strategy to promote the readiness of those involved in data collection and/or program evaluation is the use of a competency-based framework. This may be especially helpful when working with non-specialist teams, where training and exposure to research and monitoring and evaluation may vary considerably. A competency-based approach also builds on other programs developed to support non-specialist readiness to deliver scalable mental health interventions. For instance, the Enhancing Assessment of Common Therapeutic Factors (ENACT) is a globally implemented tool which forms the basis for the World Health Organization’s (WHO) Ensuring Quality in Psychological Support (EQUIP) platform (Kohrt et al., 2015a,b, 2020). ENACT is a role-play-based assessment approach that provides a competency evaluation scale for trainers’, supervisors’, and peers’ use when working with non-specialist providers of mental health support (Kohrt et al., 2015a,b). This tool, along with the EQUIP platform, was designed to set the minimum competency criteria to assure safety and enhance intervention effectiveness. For instance, EQUIP’s competency-based training was more effective compared to standard training methods, which resulted in better skill acquisition and practical implementation of task-sharing mental health interventions in a study conducted in Lebanon (Jordans and Brandon, 2020). Competency based frameworks also have emerged in the field of public health education with institutions like the Centers for Disease Control (CDC) outlining competencies for public health professionals in various fields, including community health. However, these competencies largely emphasize practice as opposed to research skills (Core Competencies for Public Health Professionals, 2023). Nonetheless, the field would greatly benefit from specific tools that identify, measure and ensure specific data collection competencies in mental health research settings.

To begin to address this gap, we developed a novel competency-based framework for the assessment and training of data collectors interacting directly with participants in interview-based encounters named the Assessment of Key Interviewing Factors in Research Assistants (AKIRA). AKIRA is an adaptable, 10-item competency-based assessment tool specifically designed to facilitate self-report interviews. AKIRA was developed in the context of the National Institutes of Mental Health (NIMH)-funded research study “Restoring mental health after COVID-19 through community-based psychological services in NYC (RECOUP-NY)”. RECOUP-NY aims to evaluate the effectiveness of Problem Management Plus (PM+) (World Health Organization, 2016), a task-sharing manualized psychological intervention for distressed adults developed by the WHO, in improving the mental health of COVID-19-impacted communities in New York City. The project focuses on training staff at community-based organizations to deliver PM+ and integrate culturally sensitive, evidence-based mental health support into existing systems of care, reducing barriers and empowering communities (RECOUP-NY, 2021).

The goal in conceptualizing AKIRA was to create a competency-based framework to ensure consistency and quality among individuals involved in data collection processes given the variability of data collectors’ experiences in research and participant interactions prior to working on this trial. The development of AKIRA is guided by the multi-step process used in the development of EQUIP and ENACT (Kohrt et al., 2015a,b, 2020). First, a careful review of the ENACT scale and EQUIP resources was conducted to ensure that the competencies found in AKIRA were adapted to be specific to data collection interviews, not overlapping with those of therapeutic nature from EQUIP tools. Second, an extensive literature review was carried where we further identified the gap in specific tools and frameworks to train non-specialists in data collection within the health field generally, and in community mental health research particularly. Third, an iterative process was used in which the conceptual development of AKIRA was informed through feedback and interviews with Key Informants and users. Fourth, AKIRA was pilot tested for feasibility and acceptability and is currently undergoing further evaluation. Once complete, a psychometric evaluation will be conducted with AKIRA.

AKIRA may be used as a framework to augment best practices, foster cross-cultural and trauma-informed communication skills, ensure fidelity in data collection, and identify harmful behaviors in the context of data collection. Moreover, it spotlights specific areas of improvement which helps to focus training, thus contributing to the overall quality and reliability of the data collected. Competencies and content included in the tool were informed by pilot testing, focus group discussions, interviews, and adaptations from other competency frameworks (e.g., ENACT; Kohrt et al., 2015a,b), as well as evidence-based and informed practices extensively documented in public and global mental health literature (e.g., Bedoya et al., 2016; Hoge et al., 2015; Malekzadeh et al., 2020; Matovu et al., 2013; Pedersen et al., 2022).

The AKIRA competency evaluation is conducted in the context of a semi-structured role-play in which an individual acts as a simulated research participant, while the data collector administers a questionnaire in a mimicked real-life interaction with an actor. Although the term “actor” is used, the individual simulating the research participant can be anyone with enough knowledge or experience working with the community they are representing in portraying this research participant. The role play is recorded and a “rater,” who receives basic training in the tool’s framework and identification of competencies, then scores the interaction and provides feedback to the data collector accordingly.

Pilot implementation results will become available in future publications. A current version of the tool will be available for use and downloadable in English and Spanish. The AKIRA tool will undergo iterative development, adaptation and refinement based on the feedback and results from further research and implementation in the United States, Uganda, and Nepal. We aim to make the tool, lessons learned, adaptable versions, and further results available as a resource to teams aiming to enhance data collection quality. A focus on competencies may also lead to improved usability and frequency of data collection, as well as greater confidence in the quality of information acquired in the context of mental health programs. Importantly, such data can then be used to compare outcomes across diverse contexts and help to guide the delivery of interventions with greater precision.

Moreover, a competency-based approach may facilitate important discussions among stakeholders involved in mental health programming about ethical practices and potential concerns communities have about sharing information. As the AKIRA framework continues to evolve, it can be tailored and adapted to reflect specific histories or cultural factors to increase awareness and sensitivity among those involved in research and monitoring and evaluation in particular contexts. Community members should be invited to partner with those delivering and researching mental health programs. Participatory contribution to this development, capacity building and adaptation work, ensures the generation and dissemination of programming that are acceptable, relevant and aligned with the needs, and unique characteristics of served communities (Coulter et al., 2020). Along with the WHO’s recommendations that mental health gaps be reduced through the training of non-mental health specialists, tools like AKIRA may also help to strengthen a research and monitoring and evaluation workforce that can complement and inform the important delivery of care.
